# Experimental Investigation of the Impact of Blended Fibers on the Mechanical Properties and Microstructure of Aeolian Sand Concrete

**DOI:** 10.3390/ma17091952

**Published:** 2024-04-23

**Authors:** Yi Zhou, Hao Li, Shuyu Yu, Haolong Guo

**Affiliations:** College of Water Conservancy and Civil Engineering, Inner Mongolia Agricultural University, Hohhot 010018, China; 17802539206@163.com (Y.Z.); guo_9610@163.com (H.G.)

**Keywords:** calcium carbonate whisker, polypropylene fiber, aeolian sand concrete, compressive strength, gray entropy

## Abstract

To investigate the effect of hybrid fibers on the compressive strength of aeolian sand concrete, compressive strength tests were conducted on aeolian sand concrete with single polypropylene fibers and aeolian sand concrete with mixed polypropylene fibers and calcium carbonate whisker, and their variation rules were studied. Using scanning electron microscopy and nuclear magnetic resonance, the microstructure and pore structure of specimens were analyzed, and a mathematical model of the relationship between compressive strength and pore structure was established with gray entropy analysis. The results show that the compressive strength of hybrid fiber aeolian sand concrete first increases and then decreases with an increase in whisker content. When the replacement rate of wind-accumulated sand is 80% and the fiber content is 0.1%, the optimal volume content of whisker is 0.4%, and the 28 d compressive strength of whisker is 24.8% higher than that of aeolian sand concrete. The average relative errors of compressive strength at 7 d and 28 d are 8.16% and 7.48%, respectively, using the GM (1,3) model. This study can provide effective theoretical support for the application of calcium carbonate whisker and polypropylene fibers in aeolian sand concrete.

## 1. Introduction

Aeolian sand is a kind of ultra-fine sand formed after wind erosion and precipitation, and it is distributed all over the world [1,2,3,4,5,6]. Due to its small particle size and inherent ease of flow [7,8], sand is particularly susceptible to wind transport. Compounded by the absence of cohesive forces between sand particles, this susceptibility often leads to the formation of sandstorms. These natural phenomena can inflict significant damage upon local ecological environments. In recent years, many scholars have extensively researched the prevention and control of aeolian sand. Aeolian sand has been used as an alternative to river sand to make concrete for the construction of desert railway roadbeds and sand damage prevention projects, which is not only economical but also environmentally friendly and plays a crucial role in the restoration of the ecological environment in the region [9,10,11,12,13,14]. However, due to the loose structure and large porosity of aeolian sand itself, the mechanical properties of aeolian sand concrete (ASC) decline rapidly with an increase in mixing amount, which greatly limits the popularization and application of ASC [15,16].

Domestic and foreign studies have shown that the incorporation of fibers into concrete can significantly inhibit the initiation and expansion of cracks, thus enhancing the mechanical properties of concrete materials [17,18]. Qais [19] found that mixing steel, PVA, and nylon fibers had a positive effect; the incorporation of steel fibers delayed the formation of cracks, PVA and nylon fibers had high strength and could absorb energy from external loads, and the amide chain (-CO-NH-) of nylon fibers and its reaction with water improved the compressive strength of the sample. Wang Long [20] studied the size effect of the mechanical properties of steel–polypropylene hybrid fiber ultra-high-performance concrete and found that steel fibers play a bridging role in the crack zone while polypropylene fibers mainly act in the fracture process zone, which can effectively inhibit the expansion of microcracks and consume energy. Yang Chengjiao [21] added steel-modified polypropylene fibers to concrete and found that when small cracks appeared in concrete, steel fibers with a high elastic modulus played the best role through their adhesion and mechanical bite force with concrete. When large cracks appear, low-elastic-modulus polypropylene fibers begin to play a strengthening role. Although the above studies considered the influence of different fiber-mixing approaches on the mechanical properties of concrete, most of them used the same scale fiber (such as centimeter-grade fiber); research on the mechanical properties of different scales of fiber concrete is currently limited. Therefore, the aim of this study was to examine how the mechanical properties of aeolian sand concrete are influenced by the addition of calcium carbonate whisker and polypropylene fibers mixed with different scales.

Based on the research results of previous scholars [22,23,24,25], in line with the concept of green and high-quality development and maximizing the utilization of aeolian sand resources, this study replaced natural river sand with 80% aeolian sand by mass and incorporated 0.1% polypropylene fiber along with different volumes of calcium carbonate whisker to optimize the internal structure of aeolian sand concrete and improve its compressive strength. The effect of mixed calcium carbonate whisker and polypropylene fibers on the pore structure of aeolian sand concrete was investigated using nuclear magnetic resonance (NMR), and its microstructure was observed using field emission scanning electron microscopy (SEM). The compressive strength model of ASC was established with gray entropy correlation analysis combined with pore structure parameters. It provides effective theoretical support for the application of calcium carbonate whisker and polypropylene fibers in ASC.

## 2. Test

### 2.1. Test Materials

Inner Mongolia Jidong P·O 42.5 ordinary Portland cement was selected for the project. The main performance indicators are shown in Table 1

The fly ash selected was Hohhot Jinqiao thermal power Plant II fly ash. Its basic performance indicators are shown in Table 2

The coarse aggregate was selected from 4.75–31.5 mm continuously graded ordinary egg gravel from the gravel-crushing field around Hohhot City. The main performance indicators are shown in Table 3.

Fine aggregates are natural river sand and aeolian sand from Kubuqi Desert. The maximum particle size of natural river sand is no more than 4.75 mm. The particle size of aeolian sand mainly ranges from 30 µm to 215 µm, accounting for 94.34% of the total. The basic physical indexes of fine aggregate are shown in Table 4

Calcium carbonate whisker was made of aragonite calcium carbonate whisker made by Dongguan Wangda Plastic Co., Ltd. (Dongguan, China) Polypropylene fibers were made of short fibers produced by Tianjin Shengvanadium Technology Development Co., Ltd. (Tianjin, China) The admixture adopted a polycarboxylic acid water-reducing agent. The water reducing rate was 25%. The mixing water was ordinary tap water.

### 2.2. Mix Ratio Design

The concrete in this experiment was designed with a strength grade of C30, and 20% fly ash was added to replace cement. Additionally, calcium carbonate whisker was added at volumes of 0%, 0.1%, 0.2%, 0.3%, 0.4%, and 0.5%, along with polypropylene fibers at a volume of 0.1% (with a length of 12 mm). The water–binder ratio was set at 0.45, the water-reducing agent content was 0.46% of the total mass of the cementing material, and the mix ratios for eight groups of concrete were calculated as shown in Table 5. The properties of the fibers can be found in Table 6.

### 2.3. Test Method

#### 2.3.1. Fine Aggregate Size Distribution Test

The significant difference in particle size between natural river sand and aeolian sand necessitates the use of two distinct methods for measuring particle size.

**Figure 1:**
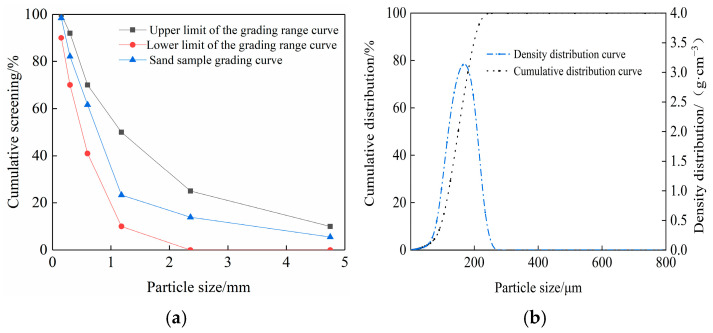
Particle size distribution of (**a**) natural river sand and (**b**) Kubuqi aeolian sand.

The natural river sand particle size distribution test was performed as follows: (1) Use a set of standard screens with pore sizes of 5, 2.5, 1.25, 0.63, 0.315, and 0.16 mm. (2) Place the 500 g dry sand sample on the shaking sieve machine and sift it successively from coarse to fine. (3) Weigh out the river sand left on each screen. (4) Calculate the particle size according to weight based on the calculation of the screening percentage and cumulative screening percentage, as shown in Figure 1a.

The aeolian sand particle size distribution test was performed as follows: Use a dry method automatic laser particle size analyzer (HL2020-L, Beijing Haixinrui Technology Co., Ltd., Beijing China) to measure the particle size distribution of aeolian sand. Weigh the appropriate amount of aeolian sand sample, pour it into the sample chamber of the laser particle size analyzer, and start measuring. The particle size distribution of the sample is analyzed according to the analysis software of the laser particle size analyzer, as shown in Figure 1b.

#### 2.3.2. Compressive Strength Test

The cube compressive strength test was carried out in accordance with the “Standard for Test Methods of Mechanical Properties of Ordinary Concrete” (GB/T50081-2019 [26]). The cube compressive strength of eight groups of concrete at the ages of 3 d, 7 d, 14 d, 21 d, and 28 d was tested using an automatic pressure testing machine (type WHY-3000, Shanghai Longhua Co., Ltd., Shanghai China). The loading speed was set at 0.5 MPa/s, and the specimens measured 100 mm × 100 mm × 100 mm. The experiment consisted of eight different mix ratios, five curing ages, and a total of 120 compressive strength cubes. Three specimens were selected from each group for cube compressive strength testing. To minimize error interference, the average of three test values was calculated, ensuring a control error of less than 15%. The formula for calculating the compressive strength of the cube is shown below.

(1) $$f_{cc}=\frac{F}{A}\times 0.95$$

The formula symbols denote the following: 

*f_cc_*—compressive strength (MPa);

*F*—specimen failure load (N);

*A*—specimen bearing area (mm^2^).

#### 2.3.3. NMR Test

In this experiment, a Meso MR23-60 (The NMR instrument was purchased by Shanghai Niumai Electronic Technology Co., Ltd., Shanghai China) nuclear magnetic resonance analysis system was used to measure the pore evolution characteristics of the specimens. During the test, the magnetic field intensity was 0.55 T, the hydrogen proton resonance frequency was 23.3 MHz, and the magnet temperature was 32 ± 0.01 °C. After standard maintenance for 24 d, the specimen was soaked in water at (20 ± 2) °C for 4 d, the specimen was sampled by a diamond core drilling machine and cutting machine, and a cylinder with a diameter of 48 mm × 50 mm was prepared. The core sample was placed in a vacuum saturation device at −0.1 MPa and filled with water for 24 h, so that the internal pores were fully filled with water, and the sample was quickly taken out and put into the nuclear magnetic resonance instrument for testing.

The transverse relaxation time *T*_2_ and porosity of concrete were determined by nuclear magnetic resonance (NMR) and CPMG sequence inversion techniques. The *T*_2_ relaxation time of nuclear magnetic resonance reflects the degree of binding and freedom of hydrogen protons in the chemical environment of a sample. For fluids in the internal pores of a material, it is generally believed that there are three different relaxation mechanisms: free relaxation, surface relaxation, and diffusion relaxation. The CPMG pulse sequence is used to collect NMR data, and the sum of the reciprocal of the three relaxation times is the reciprocal of the transverse relaxation time *T*_2_, as shown below:

(2) $$\frac{1}{T_{2}}=\frac{1}{T_{2free\;}}+\frac{1}{T_{2surface}}+\frac{1}{T_{2diffusion}}$$

where *T*_2_ is the transverse relaxation time (ms) of the pore fluid, *T*_2_ free is the transverse relaxation time (ms) of the pore fluid in a sufficiently large container, *T*_2_ surface is the transverse relaxation time (ms) caused by surface relaxation, and *T*_2_ diffusion is the transverse relaxation time (ms) caused by diffusion under a magnetic field gradient.

For concrete materials, since their pores are measured in nanometers or microns, the pores can be approximately regarded as spherical, and the pore radius can be expressed by Equation (3) as follows:

(3) $$\frac{1}{T_{2}}=\rho \times \frac{S}{V}=\rho \times \frac{3}{r}$$

where ρ is the transverse relaxation strength of concrete (μm/s), which can be calculated by =5 μm/s according to experience; S/V is the ratio of the pore surface area to volume (μm^−1^); and r is the pore radius.

The *T*_2_ spectrum obtained by NMR reflects the distribution of pore size. The transverse relaxation time is positively correlated with the pore radius, and the integral area of the *T*_2_ spectrum is approximately equal to the effective pore of concrete [27].

#### 2.3.4. Field Emission Scanning Electron Microscope Test

The surface morphologies of concrete with a curing age of 7 days and 28 days were observed by a Quanta 250 FEG scanning electron microscope from FEI company (Hillsboro, OR, USA). During the observation, the scanning electron microscope resolution was 2.0 nm, the acceleration voltage was 1 KV, and the magnification was 500~5000 times.

## 3. Results and Discussion

### 3.1. Compressive Strength of the Cube

**Figure 2:**
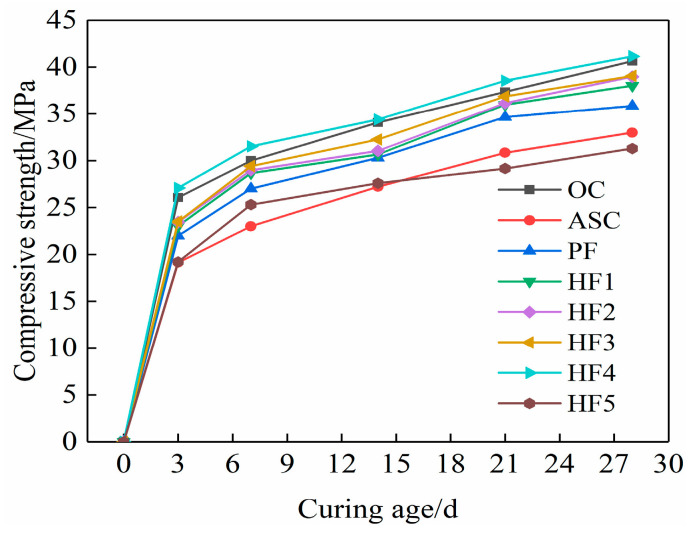
Compressive strength values at different curing ages.

Figure 2 shows the compressive strength of concrete at different curing ages. It can be seen from the figure that the compressive strength of each group of concrete test blocks shows an increasing trend with age, but the increasing amplitude is different. The compressive strength of ASC at the age of 28 d is reduced by 18.8% compared with OC, indicating that high-replacement-rate wind-accumulated sand reduces the compressive strength of concrete. The compressive strength of aeolian sand concrete (PF) mixed with polypropylene fibers is 2.9 MPa higher than that of the reference group ASC at 28 days of age, but it is still lower than OC, and the addition of an appropriate calcium carbonate whisker on the basis of PF can increase the compressive strength of concrete to varying degrees. It can be seen from Figure 2 that with an increase in whisker content from 0% to 0.5%, the compressive strength first increases and then decreases, and the compressive strength reaches the maximum value at a 0.4% whisker content (HF4), which is equivalent to OC strength at the same age. At the ages of 3 d, 7 d, 14 d, 21 d, and 28 d, the PF strength increased by 18.8%, 17.4%, 12.4%, 11.2, and 8.7% compared with ASC, respectively. The strength of HF4 was 41.4%, 36.9%, 30.8%, 25%, and 24.8% higher than that of ASC, respectively. It can be seen that the addition of polypropylene fibers can improve the compressive strength of ASC, but the addition of calcium carbonate whisker and polypropylene fibers can improve the compressive strength of ASC more significantly. This is because the multi-scale failure process of concrete materials can be described as the evolution of micropore cracking into microcracks and, ultimately, the formation of macro-cracks in the material, resulting in material failure [28]. However, polypropylene fibers can only inhibit the development of millimeter cracks but cannot inhibit micron cracks. The addition of calcium carbonate whisker makes up for the defects of fibers and can better inhibit the expansion of micron cracks. Therefore, the two fibers can play a complementary role at different scales to jointly improve ASC strength. Too much whisker content will lead to a decrease in the average distance between whiskers in the matrix [29]. When the whisker content is reduced to a certain extent, the phenomenon of whisker overlap will appear, resulting in a poor bonding effect between whiskers and concrete, causing a decrease in the strength of concrete.

### 3.2. NMR Analysis

#### 3.2.1. Pore Distribution of Hybrid Fiber Aeolian Sand Concrete

**Figure 3:**
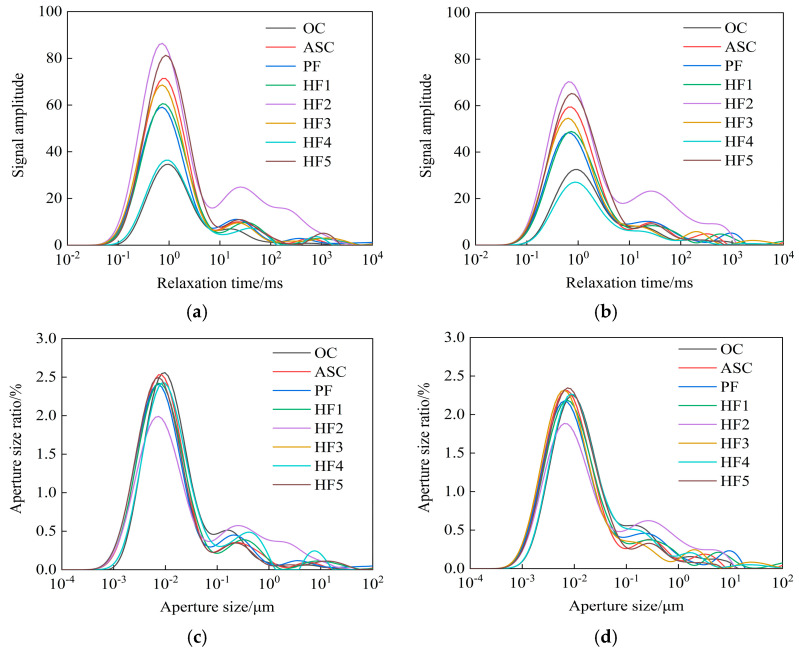
*T*_2_ spectra and pore distribution curves at different ages. (**a**) *T*_2_ atlas at 7 d of age. (**b**) *T*_2_ atlas at 28 d of age. (**c**) Pore distribution at 7 days of age. (**d**) Pore distribution at 28 days of age.

Figure 3 shows the *T*_2_ maps and pore distribution curves of concrete at 7 d and 28 d of age. According to the *T*_2_ atlas, the *T*_2_ spectrum and pore distribution of the two instars display 3~4 characteristic peaks, and the longer the relaxation time, the smaller the peak area. Using the initial relaxation time of the eight groups of concrete at 7 d of age [27], the minimum pore radii were revealed to be 1.26 × 10^−4^ μm, 8.03 × 10^−4^ μm, 7.79 × 10^−4^ μm, 6.91 × 10^−4^ μm, 5.43 × 10^−4^ μm, 3.64 × 10^−4^ μm, 2.1 × 10^−4^ μm, and 6.56 × 10^−4^ μm. The minimum pore radius of ASC first decreased and then increased when the fibers and whisker were added gradually, indicating that small pores in ASC can be developed by adding the appropriate amounts of whisker and fibers. The *T*_2_ spectral areas of the eight groups of concrete were 1615.28, 2988.87, 2754.664, 2721.893, 3392.731, 2701.338, 1504.511, and 3262.376. With an increase in whisker content, the spectral area of *T*_2_ first decreases and then increases. When the whisker content is 0.4% (HF4), the spectral area of *T*_2_ is the smallest, which also indicates that mixing an appropriate amount of whisker and fibers can optimize the internal pore structure of ASC, reduce the pore area, and make ASC denser overall. At the age of 28 d, the *T*_2_ spectral areas of the eight groups of concrete were 1448.175, 2569.683, 2220.658, 2215.387, 3105.148, 2056.368, 1193.724, and 2780.749, respectively, showing a significant decrease compared with the spectral areas at the age of 7 d. This indicates that newly generated hydration products continue to fill the pores. At the age of 28 d, the *T*_2_ spectral area of the total characteristic peak of PF and HF4 was lower than that of ASC, and the spectral area of HF4 was the smallest in the eight groups of concrete, indicating that single-doped polypropylene fibers can optimize the internal structure to a certain extent, while the effect of adding an appropriate amount of calcium carbonate whisker is more obvious.

#### 3.2.2. Pore Size Distribution of Hybrid Fiber Aeolian Sand Concrete

**Figure 4:**
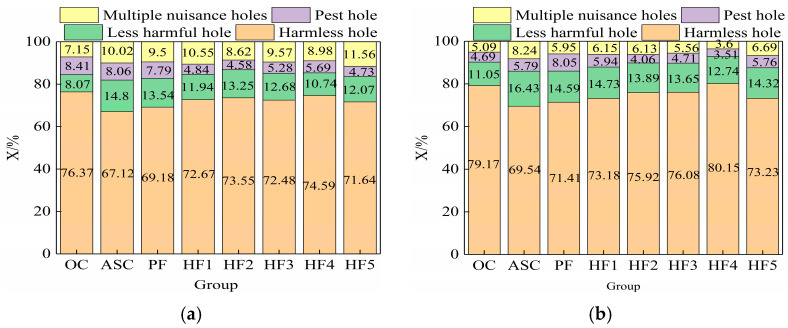
Different pore size ratios of hybrid fiber aeolian sand concrete at different ages. (**a**) The ratio of different apertures at 7 d of age. (**b**) The ratio of different apertures at 28 d of age.

**Figure 5:**
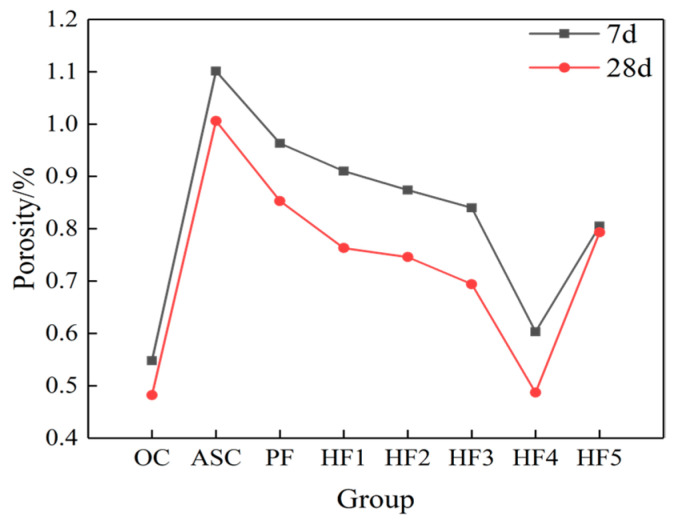
Porosity of hybrid fiber aeolian sand concrete at different ages.

According to the study by Wu Zhongwei et al. [30], aperture distribution can be divided into four types: non-harmful holes (r ≤ 0.02 μm), low-damage holes (0.02 μm ≤ r ≤ 0.05 μm), harmful holes (0.05 μm ≤ r ≤ 0.20 μm), and multi-damage holes (r ≥ 0.20 μm). Combined with the pore distribution curves at 7 d and 28 d, the pore distribution proportion and porosity curves of hybrid fiber aeolian sand concrete can be obtained, as shown in Figure 4 and Figure 5.

As can be seen in Figure 4, polypropylene fibers with a single 0.1% volume content can slightly increase the proportion of harmless holes. On this basis, with the whisker content increasing from 0% to 0.5%, the proportion of harmless holes in concrete showed a trend of first increasing and then decreasing, while the proportion of multi-damaged holes showed a trend of first decreasing and then increasing, and the proportion of harmless holes was the highest when the whisker content was 0.4% (HF4). The proportion of multi-damage holes was the lowest. At the age of 28 days, the proportion of harmless pores in HF4 was 8.74% higher than that in PF and 10.61% higher than that in ASC, and the proportion of multi-harmful pores decreased from 8.24% in ASC to 5.95% in PF and then to 3.6% in HF4. As can be seen from Figure 5, the porosity of ASC at both ages was the largest. Taking 28 d as an example, the porosity of ASC was 1.006%, and that of PF was 0.853%. With the increase in whisker content, the porosity first decreased and then increased, and the porosity of HF4 was the smallest: 0.487%. Combined with Figure 4 and Figure 5, it can be seen that with the inclusion of whisker, the porosity and the proportion of multi-damage pores both decrease and then increase, and the proportions of porosity and multi-damage pores are the smallest when the content is 0.4% (HF4). It can be seen that adding an appropriate amount of whisker and fibers can improve the internal pore structure of ASC, make the multi-damage holes in ASC develop into fewer damage holes and harmless holes, and improve the compressive strength of concrete.

### 3.3. Electron Microscope Analysis

**Figure 6:**
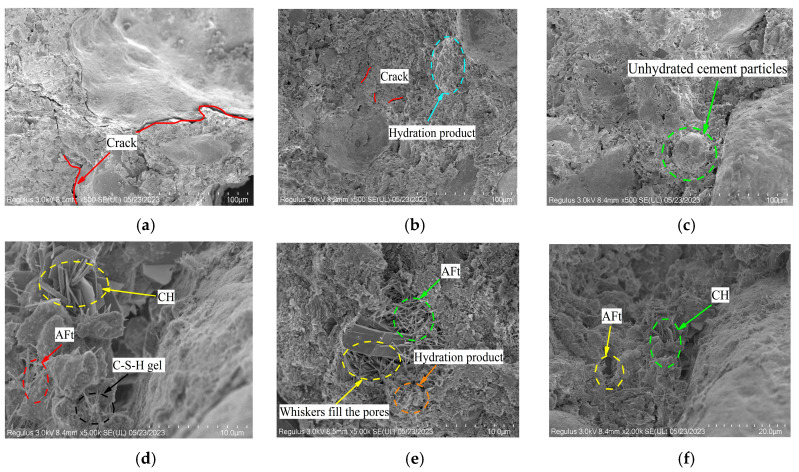
Internal micro-structure diagram under SEM. (**a**) 7 d ASC. (**b**) 7 d HF4. (**c**) 7 d OC. (**d**) 28 d ASC. (**e**) 28 d HF4. (**f**) 28 d OC.

Figure 6 shows SEM images of ASC, HF4, and OC at 7 d and 28 d of age. It can be seen from Figure 6a that at the age of 7 days, there are some micropores in ASC, and there are many small cracks [31]. Cracks and micropores are connected, forming a large area of penetrating cracks, resulting in poor mechanical properties of ASC. As can be seen from Figure 6b, although there are sporadic micropores and cracks in HF4, the cracks are not connected. The reason is that calcium carbonate whisker with a large specific surface area per unit volume can adsorb a large number of water molecules, and more hydration products are generated at the same age, which can better fill the pores and microcracks [32]. Therefore, compared with ASC, HF4 has fewer cracks at the age of 7 days, which is manifested in an improvement in macro-strength. It can be seen from Figure 6c that although there are still unhydrated cement particles in OC at the age of 7 days, there are fewer cracks and pores as a whole compared with ASC. Based on Figure 6d–f, it is evident that the three concrete groups exhibit higher densities than at 7 days. A large number of hydration products were generated, fully filling in the pores and cracks. The C-S-H gel and AFt generated inside ASC filled the internal pores without obvious cracks [31,33,34], so the strength at 28 d was significantly higher than that at 7 d. For HF4, the fibers play a bridging role, effectively inhibiting the expansion of microcracks. There are free CO_3_^2−^ ions on the whisker surface, and the cement surface is positively charged. The mutual attraction of positive and negative ions drives the cement particles to adsorb on the surface of the calcium carbonate whisker, which allows the calcium carbonate whisker and cement slurry to better bond together and fill the pores [35]. In addition, the calcium carbonate whisker can be characterized by a high modulus and strength. The concrete absorbs energy from the external load during the compression process, reducing the degree of damage [35]; however, there are only some very small pores in OC at 28 d of age, and there are no obvious large pores, so the strengths of OC and HF4 are higher.

## 4. Gray Correlation Entropy Analysis and GM (1,N) Model

### 4.1. Gray Correlation Entropy Analysis

Gray correlation analysis is a quantitative analysis of various influencing factors in a system, a comparison of the set relationship of various sequences, and an analysis of the degree of correlation among multiple factors, so as to identify the primary and secondary factors that affect research results more clearly [36].

The concrete compressive strength was taken as the system characteristic sequence *X*_0_[*x*_0_(1), *x*_0_(2), …, *x*_0_ (*k*)] (*k* = 1, 2, …, 8). The spectral area, porosity, bound fluid saturation (BFS), free fluid saturation (FFS), and the proportion of each pore interval measured by NMR were taken as the correlation factor sequence *X_i_*[*x_i_* (1), *x_i_* (2), …, *x*_i_ (*k*)] (*i* = 1, 2, …, 8). The gray correlation entropy method was used to calculate the main and secondary factors affecting the compressive strength of concrete.

### 4.2. Establishment of GM (1,3) Prediction Model

The gray GM (1,N) model is the influence of (n − 1) influencing factors on the principal variables. In this paper, the gray GM (1,3) prediction model was adopted. First, the original data needed to be dimensionalized and normalized to eliminate the impact of dimensionality on the data, and then the processed data were substituted into the model to obtain the gray correlation entropy and gray entropy correlation degree, as shown in Table 7. Finally, the concrete compressive strength, BFS, and the porosity ratio of r ≤ 0.02 μm were selected as the modeling set to establish the GM (1,3) prediction model.

The gray prediction model is as follows:

(4) $$P={\left(QTQ\right)}^{-1}Q^{T}Y$$

Among them, $Q=\left[\begin{array}{ccc} {-n}_{1}^{\left(1\right)}\left(2\right) & m_{2}^{\left(1\right)}\left(2\right) & m_{3}^{\left(1\right)}\left(2\right)\\ \vdots & \vdots & \vdots \\ {-n}_{1}^{\left(1\right)}\left(7\right) & m_{2}^{\left(1\right)}\left(7\right) & m_{3}^{\left(1\right)}\left(7\right) \end{array}\right]$,$\;Y=\left[\begin{array}{c} m_{1}^{\left(0\right)}\left(2\right)\\ \vdots \\ m_{1}^{\left(0\right)}\left(7\right) \end{array}\right]$, where: *n*_1_ is the generation sequence of compressive strength; *m*_2_ is the saturation of bound fluid from standard curing to the corresponding age; *m*_3_ is the porosity ratio of r ≤ 0.02 μm from standard curing to the corresponding age; and Y is the set of strength values from the implementation of standard curing to the corresponding age. The compressive strength data obtained from the test were used as the verification set and substituted into the GM (1,3) model to obtain the prediction model of concrete compressive strength, as shown in Equations (5) and (6).

(5) $${\hat{m}}_{1}^{\left(0\right)}\left(k\right)=-1.454n_{1}^{\left(1\right)}\left(k\right)-0.348m_{2}^{\left(1\right)}\left(k\right)+1.844m_{3}^{\left(1\right)}\left(k\right)$$

(6) $${\hat{m}}_{1}^{\left(0\right)}\left(k\right)=-1.566n_{1}^{\left(1\right)}\left(k\right)-3.576m_{2}^{\left(1\right)}\left(k\right)+5.171m_{3}^{\left(1\right)}\left(k\right)$$

Finally, the simulated value was compared with the test value, and the relative error between the two was obtained, as shown in Table 8. As can be seen from the table, the average error of the two instars is 8.16% and 7.48%, respectively, indicating that the model has high accuracy. Therefore, the pore ratio of BFS and r ≤ 0.02 μm can be used to predict the compressive strength of HF.

## 5. Conclusions

(1) The addition of low-cost calcium carbonate whisker and polypropylene fibers to high-content aeolian concrete can enhance its density and compressive strength. From an economic perspective, this is crucial for reducing the production cost of fiber-reinforced concrete materials and promoting their practical applications in engineering.
(2) The incorporation of fibers and whisker can effectively improve the compressive strength of ASC. When the replacement rate of aeolian sand is 80% and the content of polypropylene fibers is 0.1%, the compressive strength of ASC increases first and then decreases with an increase in whisker content. When the whisker content is 0.4%, its strength is the highest, which is similar to that of the OC group.
(3) When the fiber content is 0.1%, the spectral area and minimum pore radius of PF first decrease and then increase with an increase in whisker content. The addition of an appropriate amount of whisker can fill the tiny pores and optimize the internal microstructure of ASC, with the macroscopic performance demonstrating increased compressive strength.
(4) In the gray correlation analysis, the main factors affecting the compressive strength of concrete are bound fluid saturation and a pore proportion of r ≤ 0.02 μm. On this basis, a gray model GM (1,3) with compressive strength, bound fluid saturation, and r ≤ 0.02 μm pore ratio was established. The average relative errors of the predicted and tested values were 8.16% and 7.48%, respectively, indicating that the model has good accuracy. Therefore, the NMR nondestructive testing technique can be used to predict the compressive strength of concrete.

## Figures and Tables

**Table 1:** Cement performance indexes.

Fineness/%	Density/g·cm^−3^	Standard Water Consumption/%	Initial Set/min	Final Set/min	Stability	Firing Loss/%
1.4	3.12	28.5	180	365	Up to standard	1.3

**Table 2:** Basic performance indexes of fly ash.

Apparent Density/g·cm^−3^	Firing Loss/%	Moisture Content/%	Water Requirement/%	Microbead Content/%	Specific Surface Area/m^2^·kg^−1^	Fineness
2.2	3	0.8	96	93.5	350	10

**Table 3:** Basic physical property indexes of coarse aggregate.

Apparent Density/kg·m^−3^	Packing Density/kg·m^−3^	Crushing Index/%	Silt Content/%	Moisture Content/%	Particle Size Range/mm
2700	1620	3.8	0.8	0.2	4.75–31.5

**Table 4:** Basic physical property indexes of fine aggregate.

Aggregate Type	Apparent Density/kg·m^−3^	Packing Density/kg·m^−3^	Silt Content/%	Moisture Content/%	Chloride Ion Content/%	Fineness Modulus
River sand	2590	1730	1.8	2	0.28	3
Aeolian sand	2600	1375	0.4	2.2	0.03	0.6

**Table 5:** Aeolian sand concrete mix ratio (kg/m^3^).

Group	Cement	Fly Ash	Water	Gravel	River Sand	Aeolian Sand	Whisker	Fiber
OC	286	71	160	1270	654	0	0	0
ASC	286	71	160	1270	131	523	0	0
PF	286	71	160	1270	131	523	0	0.9
HF1	286	71	160	1270	131	523	2.8	0.9
HF2	286	71	160	1270	131	523	5.6	0.9
HF3	286	71	160	1270	131	523	8.4	0.9
HF4	286	71	160	1270	131	523	11.2	0.9
HF5	286	71	160	1270	131	523	14	0.9

Note: OC: ordinary concrete; ASC: aeolian sand concrete with 80% replacement rate; PF: polypropylene fiber with 0.1% volume fraction added on the basis of ASC; HF1: on the basis of PF, calcium carbonate whisker was added at a volume fraction of 0.1%, and so on.

**Table 6:** Fiber properties.

Fiber	Density	Dimensional Dimension		Mechanical Property	
		Length	Diameter	Modulus of Elasticity	Tensile Strength
Calcium carbonate whisker	2.8 g·cm^−3^	25 μm	1 μm	550 GPa	4750 MPa
Polypropylene fibers	0.9 g·cm^−3^	12 mm	75 μm	>586 MPa	>4.8 MPa

**Table 7:** Gray correlation entropy and gray entropy correlation degree of concrete.

Relevance Argument	7 d		28 d	
	Gray Correlation Entropy	Gray Entropy Correlation Degree	Gray Correlation Entropy	Gray Entropy Correlation Degree
Spectral area	2.0091	0.9662	2.0193	0.9712
Porosity	2.0199	0.9714	2.0085	0.9659
BFS	2.0739	0.9973	2.0711	0.996
FFS	2.0118	0.9675	2.0138	0.9684
r ≤ 0.02 μm	2.0731	0.9970	2.0705	0.9957
0.02 μm < r ≤ 0.05 μm	2.0352	0.9787	2.0459	0.9839
0.05 μm < r ≤ 0.2 μm	2.0664	0.9937	2.0341	0.9782
r > 0.2 μm	2.0522	0.9869	2.0646	0.9929

**Table 8:** Comparison of GM (1,3) simulation predicted value and experimental value.

t/d	Group	Experimental Value	Predicted Value	∆/%	Average Error	t/d	Group	Experimental Value	Predicted Value	∆/%	Average Error
7	ASC	0.822	0.7480	9.03	8.16	28	ASC	0.8857	0.7777	12.19	7.48
	PF	0.9651	1.1094	14.95			PF	0.9627	1.1181	16.14	
	HF1	1.0241	1.1156	8.94			HF1	1.0204	1.1088	8.66	
	HF2	1.0355	1.0803	4.33			HF2	1.0465	1.0866	3.83	
	HF3	1.0509	1.0335	1.66			HF3	1.0486	0.9932	5.28	
	HF4	1.1259	1.0591	5.93			HF4	1.105	1.0898	1.38	
	HF5	0.9047	1.0157	12.27			HF5	0.8400	0.8806	4.83	

## Data Availability

Data are contained within the article.
